# Diagnosis and Treatment of Asymptomatic Left Ventricular Systolic Dysfunction after Myocardial Infarction

**DOI:** 10.1155/2013/731285

**Published:** 2013-03-12

**Authors:** Laura Ajello, Giuseppe Coppola, Egle Corrado, Eluisa La Franca, Antonino Rotolo, Pasquale Assennato

**Affiliations:** Chair and Division of Cardiology, Policlinico Universitario “Paolo Giaccone”, Palermo, Italy

## Abstract

The increased survival after acute myocardial infarction induced an increase in heart failure with left ventricular systolic dysfunction. Early detection and treatment of asymptomatic left ventricular systolic dysfunction give the chance to improve outcomes and to reduce costs due to the management of patients with overt heart failure.

## 1. Introduction

**Figure 1:**
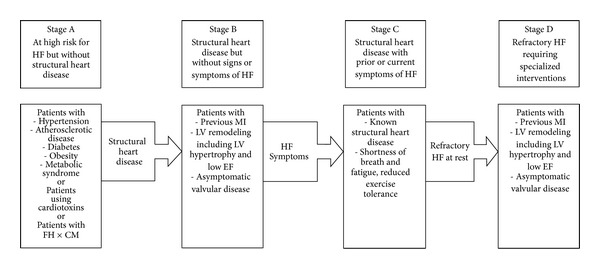
Stages of heart failure (Adapted from [3]).

Despite substantial progresses in the diagnosis and treatment of acute myocardial infarction (AMI), about 22% of men and 46% of women will be disabled with heart failure (HF) within six years [1]. About 40% of patients with an AMI develop left ventricular systolic dysfunction (LVSD) with or without signs of HF, which adversely influences quality of life, hospitalization rates, and mortality [2]. Considering the high survival rate after an AMI and the higher incidence of LVSD, early detection of people at risk of developing HF after an AMI should constitute a priority. Patients who have had an AMI, but who do not show signs of HF, could be burdened with an asymptomatic LVSD or stage B HF (Figure 1), according to ACC/AHA Guidelines of 2009 [3]. This condition is often not diagnosed and, for this reason, not treated, even if morbidity and mortality are similar to those of symptomatic HF [3]. Besides this, these patients run a higher risk because they are not aware of their pathology. Our aim is to underline the importance of an early detection of patients with asymptomatic LVSD in order to take all the measures that are necessary to reduce morbidity and mortality connected to this condition.

## 2. Epidemiology

In occidental countries, coronary heart disease (CHD) is the most important cause of LVSD and HF [4]. Ischemic cardiomyopathy is the underlying cause in about 61% of patients with signs and symptoms of HF [5]. In the SAVE trial, asymptomatic LVSD was present in 58% of patients after an AMI [6]. Robust epidemiological data about the prevalence of asymptomatic LVSD after an AMI are hard to find. Surveys indicate that only about 60% of patients with an AMI have their ventricular function assessed [7]. Hellermann et al. conducted a review of the literature between 1978 and 2000, finding that the incidence of HF was reported only in few studies and in none of these studies diagnostic criteria for assessing HF were given [8]. If the Killip classification is used, patients with asymptomatic LVSD should be classified as Killip class 1 (no evidence of pulmonary congestion or shock). Possibly the most relevant data on the incidence, prevalence, and persistence of post-MI heart failure can be derived from the TRACE study, a randomized, double-blind, and placebo-controlled study in which patients who have had an AMI were randomly assigned to receive oral trandolapril or placebo [9]. About 40% of patients from the TRACE trial developed LVSD and, among these, 74% developed clinical features of HF. Besides this, only 30% of all patients had both HF and LVSD, while 24% had features of HF in the absence of LVSD. 64% of patients developed HF or LVSD within the first few days after an AMI. TRACE results are corroborated by other population studies in which the reported incidence of HF is 22%–48%, with a mean of 37% [8]. GISSI-3 Echo substudy gives another important contribution to define LVSD soon after an AMI [10]. Using end-diastolic volume (EDV) as a marker of ventricular remodeling, the authors noted that

1. at 24–48 hours from symptoms onset and at hospital discharge, EDV decreased in 26% of patients, was stable in 23%, and increased slightly in 32%. Nineteen percent of patients showed a >20% increase at hospital discharge (severe early dilation);
2. in the period between hospital discharge and six months after AMI, EDV decreased in 31% of patients, was stable in 25%, and slightly increased in 26%. Sixteen percent of patients showed a >20% increase at six months (severe late dilation);
3. in-hospital left ventricular enlargement is not predictive of subsequent dilation and dysfunction, while late remodeling is associated with progressive deterioration of ventricular function [10]. The QRS complex changes after AMI have been correlated with infarct size and left ventricular function; by contrast, the significance of T waves changes is not clear. GISSI-3 study showed that normalization of negative T waves during the followup was more correlated with the resolution of wall motion abnormalities than QRS changes; the absence of resolution or the late appearance of new negative T waves predicts remodeling with progressive deterioration of left ventricular function [11].

## 3. Prognosis

Patients with HF and LVSD have a higher risk of adverse events (cardiac arrest, myocardial rupture, stroke, prolonged hospitalization, ventricular arrhythmias, re-AMI, and sudden death) than patients who have had an AMI but did not develop LVSD or HF [12, 13]. In the SAVE trial, authors enrolled patients with AMI and asymptomatic LVSD that were followed up for an average of 42 months. About 16% of patients, who survived after an AMI and with an ejection fraction (EF) = 40%, developed clinical features of HF. Besides this, 16% of patients in the placebo group had a deterioration of EF of 9 or more units. Mortality rate in the placebo group was 25% (12% within the first year) [14]. Also VALIANT trial showed that LVSD after an AMI is correlated to a higher incidence of sudden death. More than a half of deaths classified as sudden death or cardiac arrest happened among survivors of AMI with an EF = 30% [15]. 

## 4. Physiopathology

**Figure 2:**
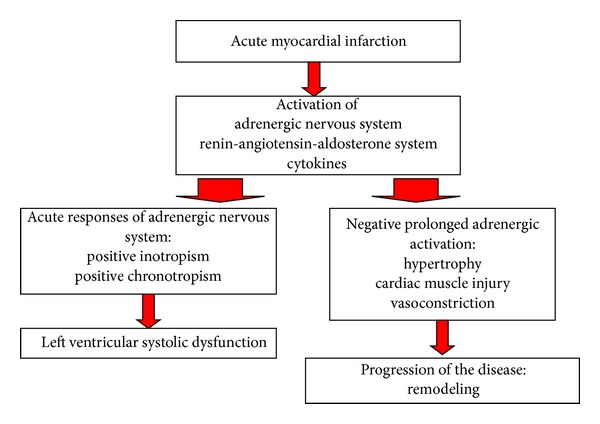
Physiopathology of left ventricular systolic dysfunction after myocardial infarction.

In LVSD following AMI, the AMI is the leading cause of the deterioration of contractility and the decrease of EF (Figure 2). The prototypic pathways that participate in maintaining blood pressure and cardiac output include the autonomic nervous system, renin-angiotensin-aldosterone system, and cytokine cascades. In addition to the positive cardiac effects on stabilizing myocardial performance, increased myocardial adrenergic signaling can lead to further cardiac damage [15]. Nowadays it is a common opinion that norepinephrine, angiotensin II, endothelin, aldosterone, and tumor necrosis factor contribute to worsen LVSD and to the passage from stage B to stage C of HF. The enlargement of left ventricle associated to the modification of its shape that also involves normal myocardial segments causes an increase of left ventricular end-diastolic wall stress (afterload): this results in the augmentation of the stroke work and in the decrease of cardiac output. A high left ventricular end-diastolic pressure in patients with asymptomatic LVSD is related to a lower EF and is an independent predictor of mortality and/or clinically overt HF [6]. So, the blockade of neurohormonal activity may reverse ventricular remodeling, preventing progression to symptomatic HF [16–18]. Other risk factors, specific for the patient (such as older age, diabetes, and hypertension) can partecipate to the process of left ventricular remodeling after an AMI [19, 20].

## 5. Diagnosis

**Figure 3:**
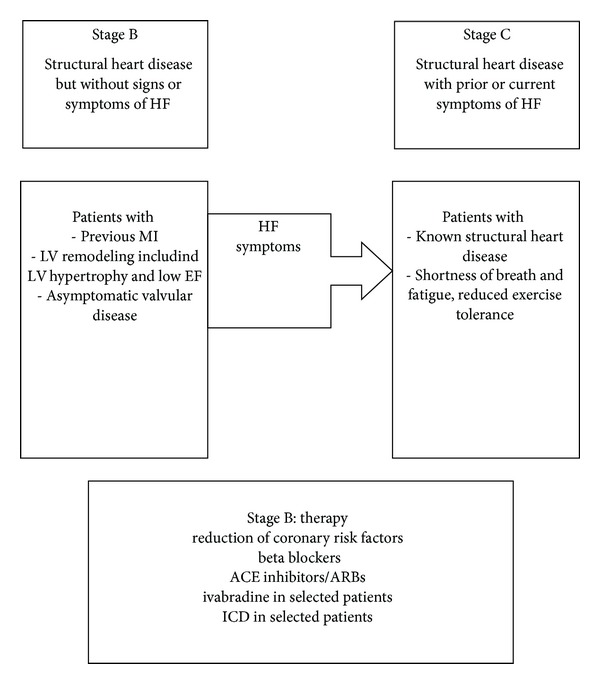
Pharmacological and nonpharmacological therapies of stage B heart failure (Adapted from [3]).

The absence of clinical signs and symptoms of HF after AMI may reflect the delay in the process of diagnosis and therapeutic measures. For this reason, an early recognition of this condition is important. The rationale is twofold. First of all, randomized studies showed that an appropriate therapy can improve significantly the prognosis. SOLVD prevention trial showed that the angiotensin-converting-enzyme inhibitor enalapril significantly reduced the incidence of deaths, hospitalization rate, and HF, as compared with the rates in the group given placebo (30% and 39%, resp.), among patients with asymptomatic LVSD and this treatment for 3-4 years led to an improvement of survival in a 12-year followup [21, 22]. Secondly, the prognosis is worse if the therapy is started on later (Figure 3). SOLVD treatment trial, despite confirming the benefit of enalapril treatment compared to placebo, showed that mortality rate at two years was 20% versus 25% in the placebo group [23]. Hence, the importance of assessing LVEF after an AMI, which is also a quality indicator in the management of AMI [20]. Nowadays the echocardiography is the method of choice for the assessment of left ventricular function. The limit of LVEF (=35% or =40%) for the identification of LVSD is still a matter of debate, even if the inverse relationship between mortality and the deterioration of LVEF is demonstrated [9, 14, 22]. Even though it cannot be considered a surrogate of echocardiography, the determination of brain natriuretic peptide (BNP) can be useful for the diagnosis of HF and to predict LVSD and left ventricular remodeling after AMI [24, 25]. However the therapeutic benefit is strongly related to an LVEF = 35–40%. 

## 6. Therapy

Current therapeutic approach to the preservation of left ventricular function in AMI consists, on one side, in reducing infarct size through the administration of ASA and, on the other side, in decelerating or preventing left ventricular remodeling and the deterioration of LVEF through the administration of angiotensin-converting-enzyme inhibitors and *β*-blockers [26–29]. This therapeutic strategy is also associated with a higher survival rate [9, 14, 18, 21, 30, 31]. 

### 6.1. ACE Inhibitors

Two important clinical trials, SAVE and TRACE, demonstrated that long-term ACEi treatment, if started early after AMI in patients with asymptomatic LVSD, could prevent progression to overt symptoms and improve survival. In the SAVE study survivors of AMI were followed up for an average of 42 months: the reduction in the risk of death from all causes in the group treated with captopril was 19%, the reduction in the risk of death due to cardiovascular causes was 21%, and the reduction in the risk of developing overt HF was 37% [14]. In the TRACE study, over 2–4 years, there was a reduction of the overall mortality rate (22%), of sudden death (24%), and of progression to overt HF (29%) [9]. Several studies have evaluated the efficacy of ACEi in patients in the acute phase of a myocardial infarction. The efficacy of this strategy seems to be strictly related to reperfusion therapy. SMILE study demonstrated that the early administration of zofenopril to patients with anterior AMI who were not receiving thrombolytic therapy significantly reduced the combined endpoint of death and severe HF at six weeks from 10.6% to 7% [32]. Interestingly the benefits of this short-term treatment were maintained over time, with an improved survival at one year. A meta-analysis of 845 patients with anterior AMI could not demonstrate regression of left ventricular dilation in patients receiving thrombolysis by ACEi treatment, while very early treatment with ACEi has a beneficial effect in patients in whom reperfusion therapy failed [33].

### 6.2. Angiotensin-Receptor Blockers (ARBs)

No clinical trials using these agents in patients with LVSD have yet been reported. However, on the basis of studies such as CHARM-Alternative and Val-HeFT and of pathophysiological and clinical aspects, ARBs should be considered in patients intolerant to ACEi. Combination therapy with an ACEi and an ARB is not currently recommended in this category of patients [34].

### 6.3. Beta Blockers (BB)

BB are administrated to the majority of patients after AMI, independently of the evaluation of LVSD, because they are safe and effective in improving survival rate and reducing the incidence of sudden death and reinfarction. Their efficacy on morbidity and mortality in patients with asymptomatic LVSD is not clear. The CAPRICORN randomized trial showed that, in patients treated a long term after AMI complicated by LVSD, carvedilol in combination with ACEi compared to placebo groups reduced the frequency of all-cause (12% versus 15%, *P* = 0.03) and cardiovascular mortality and recurrent, nonfatal myocardial infarction [18]. A retrospective analysis of data from the SAVE study showed that the use of BB was significantly associated with lower 1-year cardiovascular mortality (13% versus 22% in patients without BB) and lower occurrence of severe HF (17% versus 23% in patients without BB) [30]. REVERT study demonstrated that BB therapy can ameliorate left ventricular remodeling in patients with asymptomatic LVSD [35]. In the end, BB and ACEi are probably effective also in elderly patients with asymptomatic LVSD, even if these patients are less likely to receive this combined therapy. A retrospective analysis of patients aged ≥ 65 years old showed beneficial effects of BB in combination with ACEi among elderly patients with asymptomatic LVSD if compared to patients who received BB or ACEi or none [36].

### 6.4. Aldosterone Antagonists

Even though aldosterone antagonists are effective in reducing morbidity and mortality in patients with overt HF, they are not currently recommended in patients with asymptomatic LVSD [34]. Ongoing trials are evaluating their benefit in decelerating the process of left ventricular remodeling.

### 6.5. Digoxin

DIG study showed that digoxin did not reduce the overall mortality but reduced hospitalization rate and symptoms of HF [37]. Thus, it is not recommended in asymptomatic patients with LVSD, also because of its proarrhythmic effects, especially in women [38].

### 6.6. Other Drugs

No studies evidenced the beneficial effects of diuretics, nitrates, and calcium channel blockers in patients with asymptomatic LVSD following AMI. BEAUTIFUL trial showed no advantages in terms of cardiac outcomes in the use of ivabradine in patients with chronic ischemic cardiomyopathy, asymptomatic LVSD (LVEF < 40%), and history of myocardial infarction; on the other hand, it could be administered to reduce the incidence of coronary artery disease outcomes in a subgroup of patients who have heart rates ≥ 70 bpm [39].

### 6.7. Nonpharmacological Therapy

Although drug therapy is effective in the majority of patients in decreasing mortality rate, the risk of death in patients with LVSD is still very high. This category is burdened with a mortality rate at 4-5 years of 20% [40]. MADIT-II enrolled 1232 patients (461 asymptomatic) with a prior myocardial infarction and LVEF ≤30%; these patients were randomly assigned to receive an implantable defibrillator or conventional medical therapy. Prophylactic implantation of a defibrillator improved survival with a reduction in 20-month mortality rate of 31% [41]. Asymptomatic patients got the same beneficial effects as symptomatic patients. Another trial, the DINAMIT, in patients with a prior AMI, LVEF = 35%, and depressed heart rate variability, showed that prophylactic ICD therapy does not reduce overall mortality in high-risk patients who have recently had a myocardial infarction. Although ICD therapy was associated with a reduction in the rate of death due to arrhythmia, that was offset by an increase in the rate of death from nonarrhythmic causes [42]. Current European Guidelines recommend the implantation of ICD in patients with symptomatic or asymptomatic LVSD (LVEF 30–35%) 40 days after AMI and 3 months after an effective reperfusion therapy [43]. The usefulness of resynchronization therapy (CRT) in patients with asymptomatic LVSD is debatable. REVERSE and MADIT-CRT studies suggest that it has no efficacy in patients in NYHA functional class I-II. Thus, it is not currently recommended in this group of people [44, 45]. 

Patients with LVSD following AMI are at high risk of adverse events and, for this reason, therapy at discharge should be optimized. Data from the National Registry of Myocardial Infarction (NRMI) demonstrated that patients who suffered from HF during stay in hospital underwent less frequently the appropriate therapeutic management [46]. All the preventive measures should be taken into account in order to avoid anatomical and pathophysiological modifications that can lead to overt HF. “Overt HF should be considered a defeat rather than the first indication to treat” [47].
